# An Account of the Practice of One of the Physicians of the Westminster General Dispensary, and of the Western Dispensary, from the 20th of July, to the 20th of August, 1807

**Published:** 1807-09

**Authors:** Samuel Fothergill

**Affiliations:** Southampton Street, Strand


*An Account of the Practice of one of the Physicians of the Westminster General Dispensary, and of the Western Dispensary, from the 20th of July, to the 20th of August,* 1807.

| ACUTE DISEASES. | | | |
|---|---|---|---|
| Synochus - - - | 8 | Inflammatory Sore Throat | 3 |
| Apthous Sore Throat | 2 | Measles - - - | 2 |
| | | Cholera - - - | 3 |

Catarrh

| | | | |
|---|---|---|---|
| Catarrh - - - | 2 | Dyspepsia - - | 7 |
| Acute Rheumatism - | 4 | Gastrodynia - - | 5 |
| Erysipelas - | 1 | Enterodynia - - | 3 |
| Tertian - - - | 1 | Colica Pictonum - | 1 |
| Acute Diseases of Infants | 11 | Bilious Vomiting - | 3 |
| CHRONIC DISEASES. | | Diarrhœa - - - | 10 |
| Asthenia - - - | 18 | Jaundice - - | 1 |
| Pulmonary Consumption | 4 | Colic - - - | 2 |
| Scrophula - - | 2 | Ascites and Anasarca | 4 |
| Tabes Mesenterica - | 1 | Cephalalgia and Vertigo | 3 |
| Cough and Dyspnœa | 7 | Hydrocephalus Internus | 1 |
| Asthma - - - | 2 | Worms - - - | 3 |
| Hæmoptoe - - | 3 | Cutaneous Diseases - | 6 |
| Pleurodyne - - | 3 | Amenorrhœa - - | 2 |
| Chronic Rheumatism | 9 | Menorrhagia - - | 3 |
| Lumbago - - | 6 | Dismenorrhœa - | 2 |
| Paralysis - - | 2 | Leucorrhæa - - | 4 |

Since the last Report I have not seen a single case of typhus; synochus has frequently appeared, without any thing peculiar or extraordinary in its symptoms. The convalescence of one case, indeed, is attended with a remarkable and distressing prostration of strength; the faculties of the mind are impaired, and a general stupor prevails, more than the severity of the complaint gave reason to apprehend. The bowels were considerably purged during the continuance of the fever; several evacuations taking place daily without the operation of medicine. Change of air would probably restore the strength of the patient sooner than it could be effected by any other means. When, after acute diseases, the subsequent debility continues long, and resists the usual tonic remedies, it is to be feared that some other disease will soon destroy the broken constitution, and a journey into the country should be urged, as a likely means of promoting cheerfulness, by affording a succession of pleasing ideas, and at the same time effecting a gradual restoration of strength.

In acute diseases, from the urgency of the danger, and the dread of immediate dissolution, the patient is generally pretty tractable, and willing to submit to whatever his physician proposes for his relief; there is scarcely time to question his authority, or to doubt his practice. But in chronic cases we have often much difficulty to encounter. Every one dabbles a little in medicine, and knows something of his own case; we are called upon to account for every symptom, and to give a plausible theory of the disease; while, if we do not promise more for the effect of our medicines dicines than experience authorises, it is very likely they will not be taken. Many physicians, standing on the vantage ground of age, skill, and reputation, boldly refuse to answer all interrogation; but this is not practicable in all cases, nor with all men; and I believe it will generally be found most advantageous, where the patient is rational, to explain what we are doing; and when he cannot hear reason, to tell him something which he will not comprehend. In complaints of long continuance it requires great skill in a medical practitioner not to be dismissed before his remedies have had any beneficial effect; the patient becomes weary, and doubts the success of the proposed plan; another practitioner is called in, and for similar reasons is equally unsuccessful; the complaint in the mean time goes on, begins to be regarded as incurable, and the regular professors of medicine to be contemned. A quack is next resorted to, and now and then has the luck to restore what has been erroneously considered a lost case; and thus the most impudent and imposing traffic in the credulity, misfortune, and ignorance of mankind, that can disgrace humanity, is affirmed and upheld.

The mischief resulting from the general diffusion of empirical nostrums through the country, is only surpassed by the pestiferous effects of the numerous gin-shops which disgrace every street in London. It is a severe libel on human nature, that government derives an immense revenue from three sources, of which the fatal and morbid influence upon society is incalculable; I need scarcely name them; patent medicine warehouses, gin-shops, and lottery-offices obtrude themselves on our notice, and excite the commiseration of the philanthropist in every part of the metropolis, whilst we are sending missionaries to enlighten the uncivilized Indians, who are much more happy and innocent than a great part of our population at home. When a man has swallowed a potent quack medicine, though he may say to any officious expostulator:

> "Quid enim? concurritur: horæ

> "Momento aut cita mors venit, aut victoria læta."

It were better to remember,

> "Improvisa lethi
> "Vis rapuit rapiet que gentes."

SAMUEL FOTHERGILL.

*Southampton Street, Strand, August* 25, 1807.

